# Quantitative Assessments of Tumor Activity in a General Oncologic PET/CT Population: Which Metric Minimizes Tracer Uptake Time Dependence?

**DOI:** 10.2967/jnumed.123.266469

**Published:** 2024-09

**Authors:** Semra Ince, Richard Laforest, Malak Itani, Vikas Prasad, Paul-Robert Derenoncourt, John P. Crandall, Saeed Ashrafinia, Anne M. Smith, Richard L. Wahl, Tyler J. Fraum

**Affiliations:** 1 Department of Radiology, Washington University School of Medicine, St. Louis, Missouri;; 2 Siemens Medical Solutions Inc., Knoxville, Tennessee; and; 3 Department of Radiation Oncology, Washington University School of Medicine, St. Louis, Missouri

**Keywords:** Patlak slope, PET, corrected SUV, metabolic rate

## Abstract

In oncologic PET, the SUV and standardized uptake ratio (SUR) of a viable tumor generally increase during the postinjection period. In contrast, the net influx rate (*K_i_*), which is derived from dynamic PET data, should remain relatively constant. Uptake-time-corrected SUV (cSUV) and SUR (cSUR) have been proposed as uptake-time-independent, static alternatives to *K_i_*. Our primary aim was to quantify the intrascan repeatability of *K_i_*, SUV, cSUV, SUR, and cSUR among malignant lesions on PET/CT. An exploratory aim was to assess the ability of cSUR to estimate *K_i_*. **Methods:** This prospective, single-center study enrolled adults undergoing standard-of-care oncologic PET/CT. SUV and *K_i_* images were reconstructed from dynamic PET data obtained before (∼35–50 min after injection) and after (∼75–90 min after injection) standard-of-care imaging. Tumors were manually segmented. Quantitative metrics were extracted. cSUVs and cSURs were calculated for a 60-min postinjection reference uptake time. The magnitude of the intrascan test–retest percent change (test–retest |%Δ|) was calculated. Coefficients of determination (*R*^2^) and intraclass correlation coefficients (ICC) were also computed. Differences between metrics were assessed via the Wilcoxon signed-rank test (α, 0.05). **Results:** This study enrolled 78 subjects; 41 subjects (mean age, 63.8 y; 24 men) with 116 lesions were analyzed. For both tracers, SUV_max_ and maximum SUR (SUR_max_) had large early-to-late increases (i.e., poor intrascan repeatability). Among [^18^F]FDG-avid lesions (*n* = 93), there were no differences in intrascan repeatability (median test–retest |%Δ|; ICC) between the maximum *K_i_* (*K_i_*_,max_) (13%; 0.97) and either the maximum cSUV (cSUV_max_) (12%, *P* = 0.90; 0.96) or the maximum cSUR (cSUR_max_) (13%, *P* = 0.67; 0.94). For DOTATATE-avid lesions (*n* = 23), there were no differences in intrascan repeatability between the *K_i_*_,max_ (11%; 0.98) and either the cSUV_max_ (13%, *P* = 0.41; 0.98) or the cSUR_max_ (11%, *P* = 0.08; 0.94). The SUV_max_, cSUV_max_, SUR_max_, and cSUR_max_ were all strongly correlated with the *K_i_*_,max_ for both [^18^F]FDG (*R*^2^, 0.81–0.92) and DOTATATE (*R*^2^, 0.88–0.96), but the cSUR_max_ provided the best agreement with the *K_i_*_,max_ across early-to-late time points for [^18^F]FDG (ICC, 0.69–0.75) and DOTATATE (ICC, 0.90–0.91). **Conclusion:** *K_i_*_,max_, cSUV_max_, and cSUR_max_ had low uptake time dependence compared with SUV_max_ and SUR_max_. The *K_i_*_,max_ can be predicted from cSUR_max_.

## Visual Abstract

**Figure:**
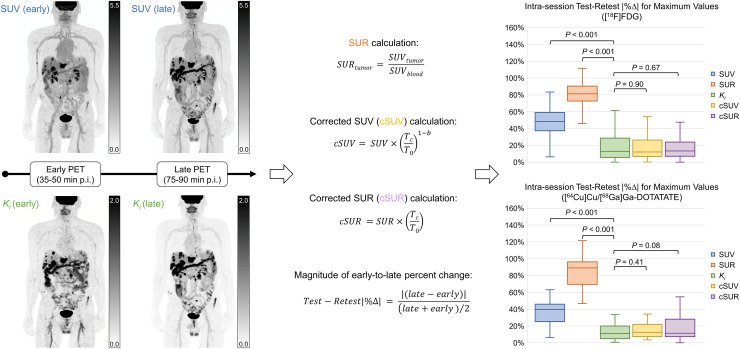

In oncologic imaging, SUV changes between scans are critical for treatment response assessment (1). However, SUV depends on uptake times, as many tumors accumulate tracer continuously (2*,*^3^). The logistic demands of busy clinical PET services often preclude precise scan-to-scan reproduction of uptake times, reducing the reliability of SUV as an oncologic biomarker (4). The Patlak model, which attempts to ameliorate this shortcoming, assumes that circulating tracer is trapped irreversibly, allowing tracer uptake to be quantified via the net influx rate (*K_i_*) (5*,*^6^). Several clinically used PET tracers, including [^18^F]FDG, approximate this behavior, permitting Patlak modeling of dynamic PET data. Once steady-state conditions are achieved between the blood and tissue compartments, the *K_i_* should remain relatively constant, whereas the SUV is expected to increase with time. Furthermore, *K_i_*-based metrics are promising prognostic biomarkers for several cancer types, occasionally outperforming SUV-based metrics (7*,*^8^).

However, *K_i_* derivation requires direct measurement or estimation of arterial input functions (AIFs) and dynamic acquisitions to generate tissue time–activity curves (5). The required modifications to PET protocols may increase imaging time or introduce motion-related quantitative errors (9). The uptake-time-corrected SUV (cSUV), which involves retrospectively modifying an observed SUV on the basis of actual versus targeted uptake times, is an alternative means of addressing the uptake time dependence of SUVs (10). Furthermore, the uptake-time-corrected tumor-to-blood standardized uptake ratio (cSUR) may allow for *K_i_* estimation, without the need for AIFs or dynamic imaging (11).

To our knowledge, no prior studies have assessed the relative temporal stabilities of SUV, SUR, *K_i_*, cSUV, and cSUR in a broad oncologic PET population. Thus, our primary aim was to quantify the intrascan repeatability of these metrics and thereby determine which approach provides the most time-independent assessment of tracer avidity on [^18^F]FDG PET and DOTATATE PET. An exploratory aim was to determine the ability of cSUR to estimate *K_i_*.

## MATERIALS AND METHODS

### Study Design

This prospective, Institutional Review Board–approved, Health Insurance Portability and Accountability Act–compliant study (NCT04283552) enrolled 78 subjects from a pool of consecutive patients scheduled to undergo standard-of-care (SOC) oncologic PET/CT for various indications, using [^18^F]FDG, [^68^Ga]Ga-DOTATATE, [^64^Cu]Cu-DOTATATE, or [^18^F]piflufolastat (note that [^68^Ga]Ga-DOTATATE and [^64^Cu]Cu-DOTATATE are hereafter collectively called DOTATATE, as these scans were analyzed together). These tracers have been reported to satisfy the Patlak model’s assumptions (12–14). The 2 [^18^F]piflufolastat studies were excluded because of insufficient cases for tracer-specific analysis. All imaging occurred at a tertiary-care center between June 2020 and October 2022. Inclusion criteria included being at least 18 y of age, having the ability to provide written informed consent, and self-reporting the ability to tolerate approximately 90 min of near-motionless supine positioning. Study imaging was performed before and after SOC PET/CT using the same tracer dose.

### Imaging Protocol

**FIGURE 1.**
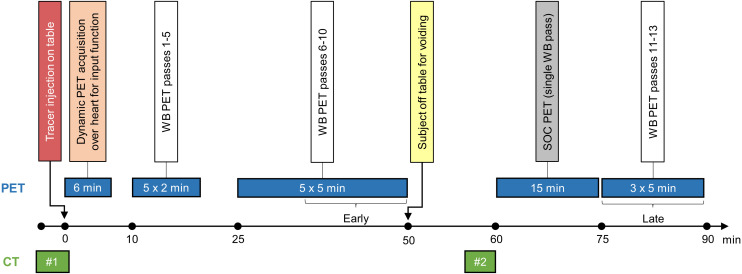
PET/CT acquisition protocol. Brackets show timing of data used for early and late image reconstructions.

The study imaging protocol (details are available in the supplemental materials; available at http://jnm.snmjournals.org) is summarized in Figure 1. All patients were imaged on a single Biograph Vision 600 PET/CT scanner (Siemens Healthineers) equipped with commercially available software for direct reconstruction of multiparametric PET images (FlowMotion Multiparametric PET Suite; Siemens Healthineers).

### PET Image Reconstruction

Using automated scanner tools, volumes of interest were placed in the descending thoracic aorta on a 6-min dynamic chest acquisition and the subsequent 10 whole-body (WB) passes (15). Per default scanner software settings, AIFs were generated from measured blood activity concentrations via exponential ([^18^F]FDG) or linear piecewise (DOTATATE) curve fitting. After all WB PET passes were reviewed dynamically for large bulk motion events, early and late SUV and *K_i_* images were reconstructed per manufacturer-recommended parameters (Supplemental Table 1). Each reconstruction used data from three 5-min WB passes with targeted acquisition times of 35–50 min (early) and 75–90 min (late) after injection. Note that the scanner software requires at least 3 WB passes for Patlak analysis. The 3 latest pre-SOC WB passes were selected for the early images, ensuring adequate time to achieve steady-state conditions. Importantly, subjects left the scanner to void immediately before SOC imaging per our standard clinical protocol, precluding automated scanner measurement of post-SOC blood tracer concentrations due to different patient positioning. Consequently, the AIF for the post-SOC *K_i_* reconstructions was automatically derived by the scanner software from extrapolation of the pre-SOC AIF (i.e., no incorporation of measured post-SOC blood tracer concentrations). SUV was based on actual body weight with units of grams per milliliter. *K_i_* had units of milliliter per minute per 100 mL. In contrast to [^18^F]FDG, intravascular DOTATATE does not enter red blood cells, requiring correction of measured *K_i_* values for the subjects’ hematocrit levels (16):

$$\text{corrected}\;K_{i}=\frac{\text{measured}\;K_{i}}{1-\text{hematocrit}}.$$

### Quantitative Analysis

Tracer-avid lesions deemed to represent sites of viable malignancy on the SOC PET/CT interpretation were selected by one author. In cases of numerous lesions, the largest or most tracer-avid lesions were selected (5 per subject maximum). Each lesion was manually segmented in MIM version 7.1.5 (MIM Software) on 4 PET image sets (*K_i_*-early, SUV-early, *K_i_*-late, SUV-late) to generate volumes of interest, using coregistered CT images for guidance. Maximum and peak values were extracted. Additionally, a cylindric volume of interest (1-cm diameter, 6-cm length) was placed in the descending thoracic aorta (avoiding vessel walls) to extract a mean value for SUR calculation:

$$\text{tumor}\;\text{SUR}=\frac{\text{tumor}\;\text{SUV}}{\text{blood}\;\text{SUV}}.$$

 SUV_max_ and SUV_peak_ were used to calculate maximum SUR (SUR_max_) and peak SUR (SUR_peak_), respectively; the SUV_mean_ of blood was used in both cases.

### Uptake Time Correction

Actual uptake time ranges were extracted for each image set, with the mid point defining the effective uptake time (e.g., 44.5 min for 37–52 min after injection). cSUV and cSUR were calculated as follows (10*,*^11^):

$$\text{cSUV}=\text{SUV}\times {\left(\frac{T_{\text{c}}}{T_{0}}\right)}^{1-b},$$

$$\text{cSUR}=\text{SUR}\times \left(\frac{T_{\text{c}}}{T_{0}}\right).$$

**FIGURE 2.**
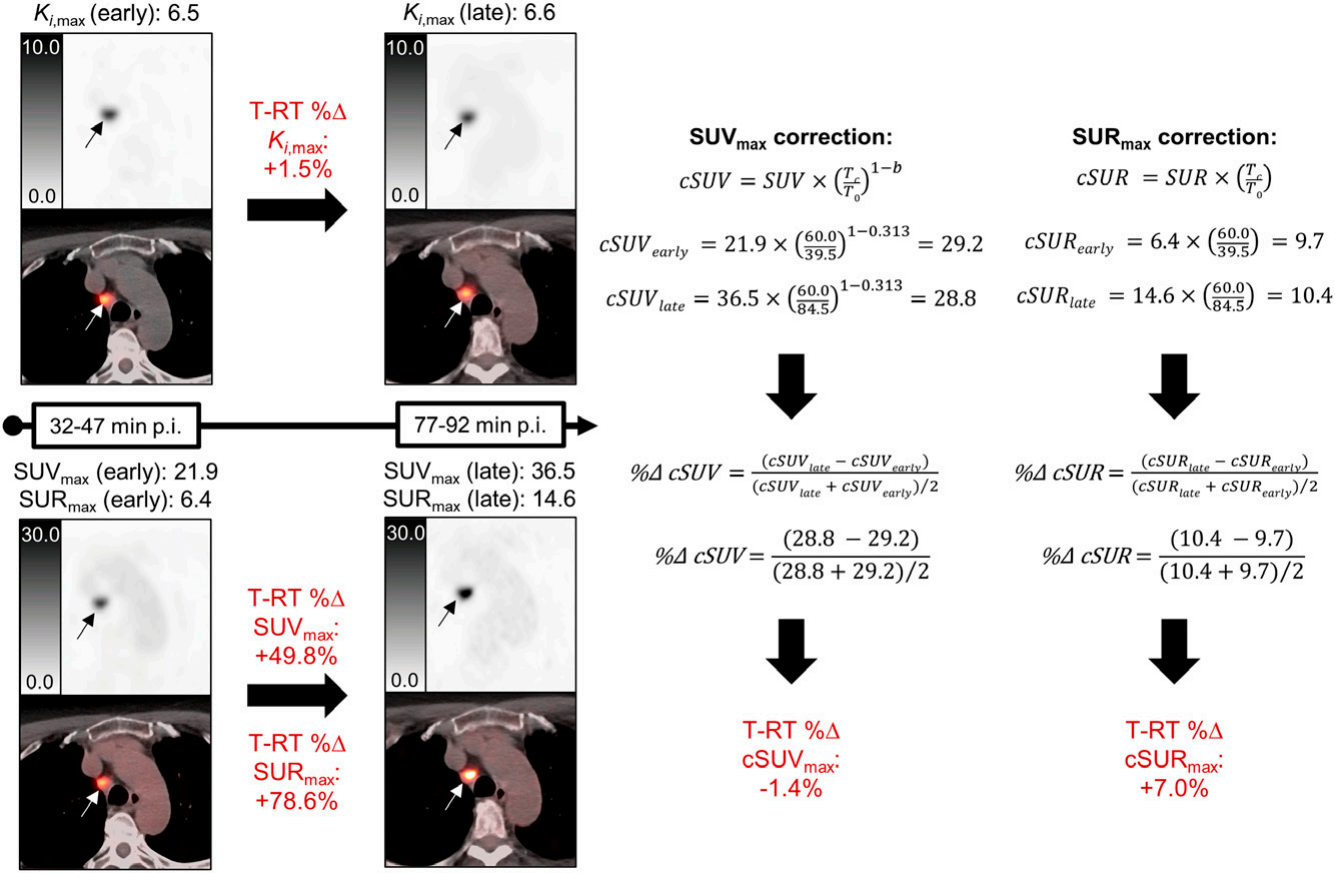
Uptake time correction procedure for SUV and SUR. Axial [^18^F]FDG PET and fused [^18^F]FDG PET/CT images are shown for *K_i_* (top) and SUV (bottom) reconstructions at early and late time points. *K_i_*_,max_, SUV_max_, and SUR_max_ of [^18^F]FDG-avid mediastinal lymph node (arrows) are shown. Test–retest %Δ values were 1.5%, 49.8%, and 78.6% for *K_i_*_,max_, SUV_max_, and SUR_max_, respectively, indicating much better intrascan repeatability for *K_i_*_,max_. Procedure for correcting SUV_max_ and SUR_max_ to 60 min after injection is also shown. Test–retest %Δ values were −1.4% for cSUV_max_ and +7.0% for cSUR_max_, similar to *K_i_*_,max_ results.

SUV and SUR are measured values, *T*_0_ is the actual uptake time, and *T*_c_ is the correction time reference. *T*_c_ was set to 60 min, reflecting a commonly targeted uptake time in [^18^F]FDG and DOTATATE protocols (1). Figure 2 shows the SUV and SUR correction procedure for a representative case. For [^18^F]FDG, the value of the parameter *b* of 0.313 was based on a prior study (10). For DOTATATE, we empirically derived a *b* of 0.63 by determining the value (averaged across all subjects) that best reproduced the observed late SUV_max_ from the observed early SUV_max_. The early and late values were then corrected to 60 min with the cSUV equation.

### Manual Patlak Analysis

To explore apparent temporal variations in *K_i_*, we selected 6 [^18^F]FDG, 1 [^68^Ga]Ga-DOTATATE, and 2 [^64^Cu]Cu-DOTATATE cases with at least 1 lesion exhibiting a large (>20% or <20%) test–retest percent change (%Δ) in maximum *K_i_* (*K_i_*_,max_) for further analysis. For all 9 cases, extrapolated AIF curve fits were compared with manually measured blood activity concentrations on the WB passes. Areas under the time–activity curve were compared for extrapolated AIF curve fits versus manual measurements via trapezoidal integration. For 4 cases, full manual Patlak analysis was performed for selected lesions and reference organs (supplemental materials) (17–20).

### Statistical Analysis

Statistical analysis was conducted in Prism 9 (GraphPad) and Excel 2016 (Microsoft) by one author with statistician guidance. Participant and scan characteristics were summarized descriptively. Because of the anticipated pharmacokinetic differences, the [^18^F]FDG cases were analyzed separately from the DOTATATE cases. The 2-tailed Wilcoxon signed-rank test was used for pairwise comparisons of quantitative metrics. Intrascan test–retest changes were computed:

test-retest Δ=late−early.

To facilitate comparisons across metrics of different magnitudes, intrascan test–retest %Δ was also computed:

$$\text{test-retest}\;\text{\%}\Delta =\frac{\text{late}-\text{early}}{\left(\text{late}+\text{early}\right)/2}.$$

Results were displayed via Bland–Altman plots and box-and-whisker plots (21). The mean (μ) and SD (σ) of the test–retest Δ and test–retest %Δ distributions were determined for each metric. The 95% limits of repeatability were defined as follows:

95% limits of repeatability=µ±2σ.

Given that near-zero %Δ values could be due to averaging of large negative and positive changes, absolute test–retest %Δ (test–retest |%Δ|) values were also computed. Intraclass correlation coefficients (ICC) and coefficients of determination (*R*^2^) were also used to assess test–retest repeatability and to quantify the accuracy of *K_i_* prediction by other metrics. A *P* value of less than 0.05 defined statistical significance. More detailed statistical methods are available in the supplemental materials (22).

## RESULTS

### Study Cohort

**FIGURE 3.**
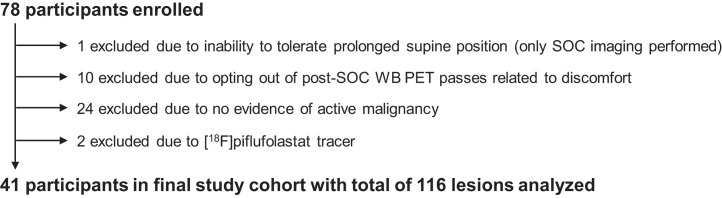
Study flowchart.

Of the 78 study subjects, 41 subjects with 116 lesions (93 on [^18^F]FDG; 23 on DOTATATE) were included in the analysis (Fig. 3). This study cohort was 58.5% men (24/41) with a mean age of 63.8 y. Additional patient and scan characteristics are captured in Supplemental Table 2.

### Intrascan Repeatability of Tumor Uptake Metrics

**FIGURE 4.**
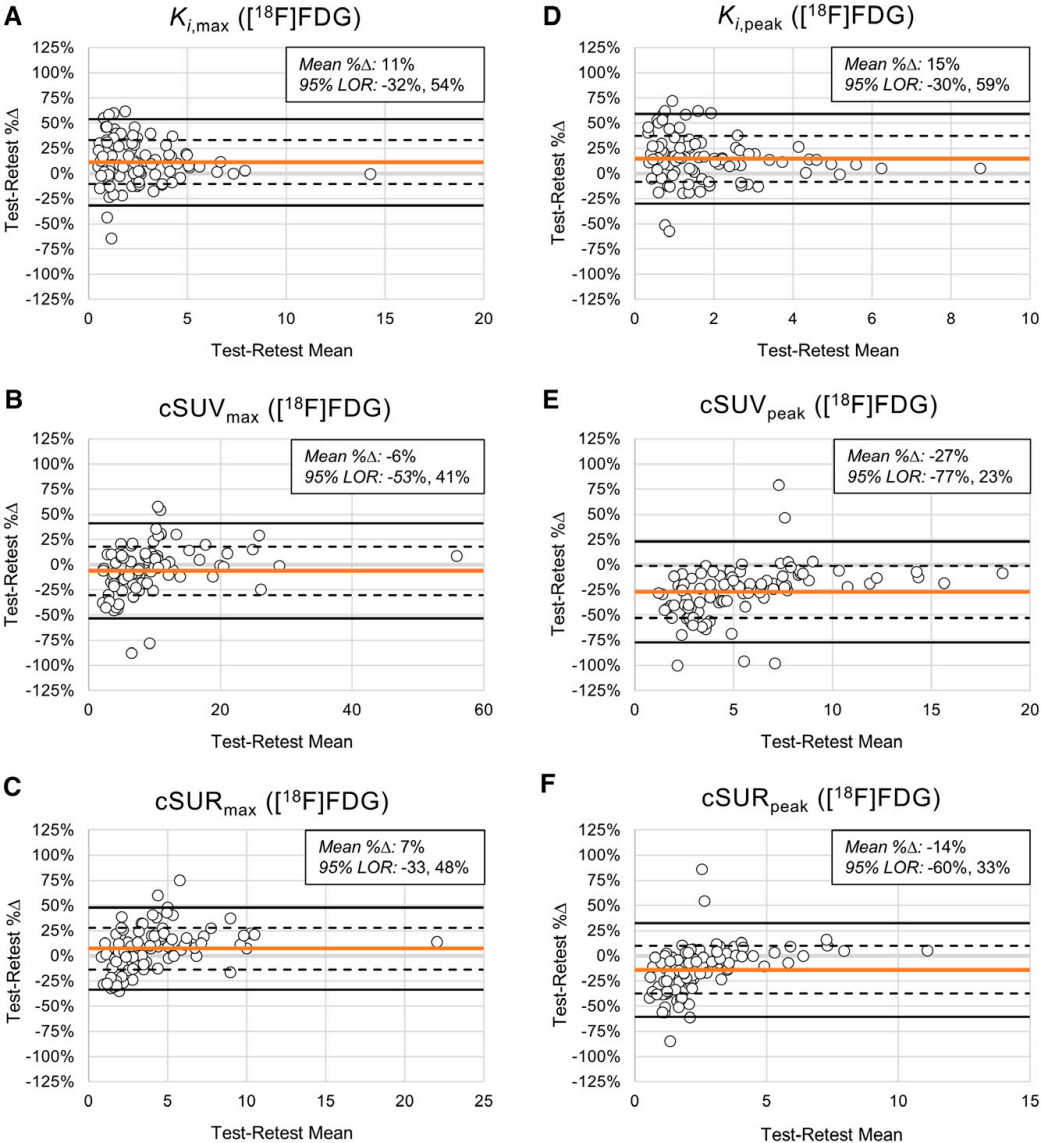
Intrascan test–retest repeatability for [^18^F]FDG-avid lesions. Bland–Altman plots are shown for *K_i_*_,max_ (A), cSUV_max_ (B), cSUR_max_ (C), *K_i_*_,peak_ (D), cSUV_peak_ (E), and cSUR_peak_ (F). Horizontal orange lines represent test–retest %Δ means. Horizontal dashed and solid black lines represent ±1σ and ±2σ for test–retest %Δ distributions, respectively. Each open circle represents [^18^F]FDG-avid lesion. 95% limits of repeatability (LOR) are computed as −2σ, +2σ.

**FIGURE 5.**
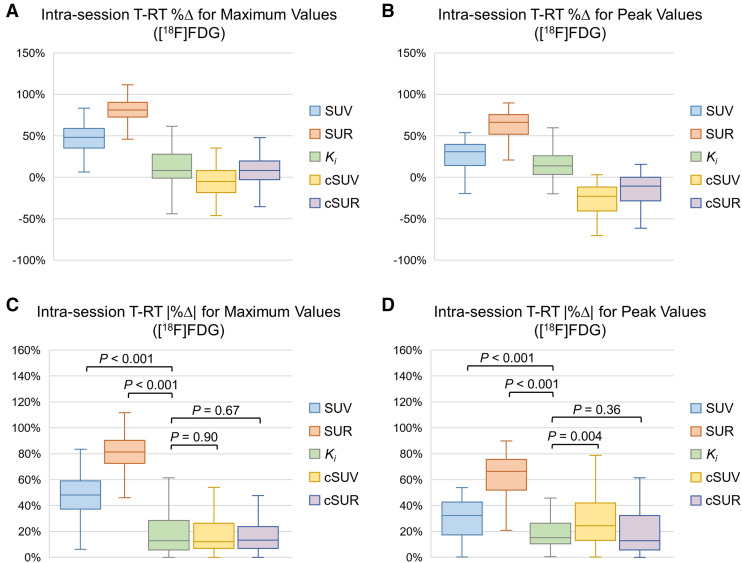
Test–retest %Δ and |%Δ| distributions for [^18^F]FDG-avid lesions. Box-and-whisker plots show test–retest %Δ (A and B) and |%Δ| (C and D) distributions for maximum (A and C) and peak (B and D) values of *K_i_*, SUV, cSUV, SUR, and cSUR. All *P* values are based on comparison to *K_i_*_,max_ or *K_i_*_,peak_. Table 2 provides descriptive statistics.

Test–retest repeatability results are summarized in Table 1 ([^18^F]FDG) and Supplemental Table 3 (DOTATATE). Scatterplots of late versus early metric values are shown in Supplemental Figures 1 and 2 ([^18^F]FDG) and Supplemental Figures 3 and 4 (DOTATATE). Bland–Altman plots for *K_i_*, cSUV, and cSUR are displayed in Figure 4 ([^18^F]FDG) and Supplemental Figure 5 (DOTATATE). Table 2 ([^18^F]FDG) and Supplemental Table 4 (DOTATATE) summarize test–retest |%Δ| values for all metrics. Box-and-whisker plots of test–retest %Δ and test–retest |%Δ| distributions are presented in Figure 5 ([^18^F]FDG) and Supplemental Figure 6 (DOTATATE). The remainder of this section focuses on the [^18^F]FDG results, except as noted (detailed DOTATATE results are provided in the supplemental materials).

**TABLE 1.** Intrascan Repeatability of SUV, cSUV, SUR, cSUR, and *K_i_* Metrics Among [^18^F]FDG-Avid Lesions

Metric	Early*	Late*	*P* ^†^	*R* ^2^	ICC	T-RT Δ^‡^	T-RT %Δ^‡^
*K_i_*_,max_ (mL/min/100 mL)	1.8 (1.1, 3.4)	2.3 (1.2, 3.5)	**<0.001**	0.96	0.97	0.2 (−0.6, 1.1)	11% (−32%, 54%)
*K_i_*_,peak_ (mL/min/100 mL)	1.2 (0.7, 2.1)	1.4 (0.9, 2.5)	**<0.001**	0.96	0.97	0.2 (−0.4, 0.8)	15% (−30%, 59%)
SUV_max_ (g/mL)	6.0 (3.9, 7.5)	9.2 (5.8, 14.5)	**<0.001**	0.93	0.64	4.9 (−4.9, 14.8)	47% (3%, 91%)
SUV_peak_ (g/mL)	3.8 (2.6, 5.7)	5.2 (2.9, 8.5)	**<0.001**	0.90	0.75	1.9 (−2.9, 6.7)	26% (−22%, 75%)
cSUV_max_ (g/mL)	8.0 (5.2, 10.1)	7.3 (4.4, 11.5)	0.17	0.92	0.96	0.0 (−4.4, 4.4)	−6% (−53%, 41%)
cSUV_peak_ (g/mL)	5.2 (3.5, 7.9)	3.8 (2.2, 6.7)	**<0.001**	0.90	0.91	−1.1 (−3.8, 1.5)	−27% (−77%, 23%)
SUR_max_ (g/mL)	2.1 (1.4, 3.1)	5.1 (3.2, 8.2)	**<0.001**	0.92	0.27	3.8 (−2.0, 9.6)	81% (48%, 114%)
SUR_peak_ (g/mL)	1.4 (1.0, 2.1)	2.8 (1.7, 4.5)	**<0.001**	0.94	0.41	1.8 (−1.4, 5.0)	63% (23%, 102%)
cSUR_max_ (g/mL)	3.2 (2.2, 4.7)	3.4 (2.3, 5.7)	**<0.001**	0.93	0.94	0.5 (−1.4, 2.4)	7% (−33%, 48%)
cSUR_peak_ (g/mL)	2.2 (1.4, 3.1)	1.9 (1.2, 3.3)	**<0.001**	0.94	0.96	−0.2 (−1.1, 0.8)	−14% (−60%, 33%)

* Values are median with first quartile and third quartile in parentheses.

† Early versus late values via Wilcoxon signed-rank test.

‡ Values are mean with 95% limits of repeatability in parentheses.

T-RT = test-retest.

Bold *P* values are statistically significant.

**TABLE 2.** Test–retest Absolute %Δ for SUV, cSUV, SUR, cSUR, and *K_i_* Metrics Among [^18^F]FDG-Avid Lesions

Metric	T-RT |%Δ|*	*P* ^†^
*K_i_*_,max_	13% (6%, 29%)	*ref*
SUV_max_	48% (37%, 59%)	**<0.001**
SUR_max_	81% (73%, 90%)	**<0.001**
cSUV_max_	12% (7%, 26%)	0.90
cSUR_max_	13% (7%, 24%)	0.67
*K_i_*_,peak_	15% (11%, 26%)	*ref*
SUV_peak_	32% (17%, 43%)	**<0.001**
SUR_peak_	66% (52%, 76%)	**<0.001**
cSUV_peak_	25% (13%, 42%)	**0.004**
cSUR_peak_	13% (6%, 32%)	0.36

* Values are median with first quartile and third quartile in parentheses.

† Comparison to *K_i_*_,max_ (rows 1–5) or *K_i_*_,peak_ (rows 6–10) via Wilcoxon signed-rank test.

T-RT = test–retest; *ref* = reference.

Bold *P* values are statistically significant.

As expected, SUV and SUR metrics showed large, statistically significant early-to-late increases. In contrast, cSUV and cSUR metrics were similar at early and late time points, though with some small but significant early-to-late changes. For the maximum cSUV (cSUV_max_), the early and late values were statistically equivalent (median, 8.0 vs. 7.3; *P* = 0.17). Surprisingly, the *K_i_* metrics exhibited significant early-to-late increases (median, 1.8 vs. 2.3; *P* < 0.001). The early and late values of each metric were strongly correlated (*R*^2^, 0.90–0.96). However, the ICCs showed substantially better agreement between early and late values for *K_i_*, cSUV, and cSUR metrics (range, 0.91–0.97) than for SUV and SUR metrics (range, 0.27–0.75). For DOTATATE, the results were similar, except that the *K_i_* metrics exhibited significant (or nearly significant) early-to-late decreases.

In the Bland–Altman analysis, cSUV_max_ and maximum cSUR (cSUR_max_) showed the least bias between early and late values, with mean test–retest %Δ values of −6% and 7%, respectively, compared with 11% for *K_i_*_,max_. In contrast, the mean test–retest %Δ values for SUV_max_ and SUR_max_ were 47% and 81%, respectively, indicating large early-to-late increases. Regarding the magnitude of deviation from perfect repeatability (i.e., test–retest |%Δ| = 0), the test–retest |%Δ| of *K_i_*_,max_ (median, 13%) was similar to those of cSUV_max_ (median, 12%; *P* = 0.90) and cSUR_max_ (median, 13%; *P* = 0.67) but significantly less than those of SUV_max_ (median, 48%; *P* < 0.001) and SUR_max_ (median, 81%; *P* < 0.001). The test–retest |%Δ| of the peak *K_i_* (*K_i_*_,peak_) (median, 15%) was significantly lower than that of all other relevant metrics except for the peak cSUR (cSUR_peak_) (median, 13%; *P* = 0.36). For DOTATATE, the results were similar to those of [^18^F]FDG for the *K_i_*_,max_ analysis, though the median test–retest |%Δ| of the *K_i_*_,peak_ was similar to that of SUV_peak_ (rather than cSUR_peak_).

### Prediction of *K_i_* by cSUR

Supplemental Figures 7 and 8 ([^18^F]FDG) and Supplemental Figures 9 and 10 (DOTATATE) show correlation results for *K_i_*_,max_ versus SUV_max_, SUR_max_, cSUV_max_, and cSUR_max_. For [^18^F]FDG, the maximum values of all 4 metrics strongly predicted the corresponding early (*R*^2^, 0.81–0.92) and late (*R*^2^, 0.81–0.91) *K_i_*_,max_ values. However, agreement with *K_i_*_,max_ by ICCs was the highest for SUR_max_ (0.94) and cSUR_max_ (0.75) at the early time point and for cSUR_max_ (0.69) at the late time point. Similarly, for DOTATATE, the maximum values of all 4 metrics strongly predicted the corresponding early (*R*^2^, 0.88–0.96) and late (*R*^2^, 0.88–0.93) *K_i_*_,max_ values. However, in contrast to [^18^F]FDG, agreement with *K_i_*_,max_ by ICCs was high for all 4 metrics at the early time point (range, 0.85–0.93) but only for cSUV_max_ (0.78) and cSUR_max_ (0.90) at the late time point.

### Manual Patlak Analysis

Supplemental Figures 11 and 12 show *K_i_*_,max_ test–retest %Δ values for each subject’s lesions for [^18^F]FDG and DOTATATE, respectively. Supplemental Tables 5 and 6 present manual Patlak analyses for several [^18^F]FDG and DOTATATE subjects, respectively. Supplemental Figures 13 and 14 capture AIF and tissue-response curves and manual Patlak plots for representative [^18^F]FDG and DOTATATE cases, respectively. Supplemental Figure 15 illustrates the effects of motion and image noise on *K_i_* and SUV. For [^18^F]FDG, the AIF curve-fit extrapolation mildly underestimated late blood activity concentrations, contributing to higher late *K_i_* values. Furthermore, motion of small lesions across WB passes contributed to *K_i_* errors that were ameliorated by manual frame-by-frame segmentations. For DOTATATE, the AIF curve-fit extrapolation moderately overestimated late blood activity concentrations, resulting in lower late *K_i_* values; additionally, DOTATATE binding appeared to be reversible at late time points for some cases. More details are provided in the supplemental materials.

## DISCUSSION

In this study, we quantified the intrascan repeatability of *K_i_*, SUV, cSUV, SUR, and cSUR metrics for [^18^F]FDG-avid and DOTATATE-avid lesions on WB PET in a general oncology population. For both tracers, SUV_max_ and SUR_max_ showed large early-to-late increases (i.e., poor intrascan repeatability). For [^18^F]FDG, there were no significant differences in intrascan repeatability between *K_i_*_,max_ (median test–retest |%Δ|, 13%; ICC, 0.97) and either cSUV_max_ (median test–retest |%Δ|, 12%; *P* = 0.90; ICC, 0.96) or cSUR_max_ (median test–retest |%Δ|, 13%; *P* = 0.67; ICC, 0.94). The intrascan repeatability of the *K_i_*_,peak_ was better than that of the peak cSUV (cSUV_peak_) but similar to that of cSUR_peak_. For DOTATATE, there were no significant differences in intrascan repeatability between *K_i_*_,max_ (median test–retest |%Δ|, 11%; ICC, 0.98) and either cSUV_max_ (median test–retest |%Δ|, 13%; *P* = 0.41; ICC, 0.98) or cSUR_max_ (median test–retest |%Δ|, 11%; *P* = 0.08; ICC, 0.94). Again, intrascan repeatability of the *K_i_*_,peak_ was better than that of cSUV_peak_ or cSUR_peak_.

Early-to-late increases are a well-known limitation of SUVs and SURs for tumor response assessments (2*,*^3^*,*^23^). As such, the Quantitative Imaging Biomarkers Alliance recommends that uptake times for baseline and follow-up scans be approximately 60 min with a no more than 10 min difference between scans (1). However, differences greater than 10 min are not uncommon. Methods to correct SUV and SUR for uptake time (i.e., cSUV, cSUR) have been published (10*,*^23^). For example, a study reported that correcting SUVs and SURs from 20 min to 55 min after injection reduced differences with actual values at 55 min from −30% to 2% for SUV and from −52% to −3% for SUR (10). This study, which used data from 9 male patients with colorectal liver metastases, proposed the simple SUV and SUR correction equations used in our work.

We verified that cSUV, using the published time parameter *b* of 0.313, is a relatively time-independent marker of tumoral [^18^F]FDG avidity. For [^18^F]FDG, our mean test–retest %Δ values of −6% and 7% for cSUV_max_ and cSUR_max_, respectively, are slightly greater in magnitude than the values cited above, possibly because of our longer early-to-late intervals or heterogeneous patient cohort. We empirically derived a *b* value of 0.63 for DOTATATE and found that cSUV is also a relatively time-independent marker of tumoral DOTATATE avidity, with mean test–retest %Δ values of 2% and −7% for cSUV_max_ and cSUR_max_, respectively. Compared with cSUV_max_ and cSUR_max_, cSUV_peak_ and cSUR_peak_ showed worse intrascan repeatability, with sizeable negative test–retest %Δ values for both tracers. The reason for this somewhat surprising finding is unclear, as peak measurements (because of their larger sampling volumes and lower potential for noise-related errors) are generally considered more repeatable than maximum measurements (24).

In terms of test–retest |%Δ| and ICC, the intrascan repeatability was similar across *K_i_*_,max_, cSUV_max_, and cSUR_max_ for both tracers. However, we observed small but statistically significant early-to-late increases and decreases in *K_i_*_,max_ for [^18^F]FDG and DOTATATE cases, respectively. In contrast, cSUV_max_ and cSUR_max_ showed no significant early-to-late changes for either tracer, with the exception of a small significant increase in cSUR_max_ for DOTATATE. For both tracers, the observed early-to-late *K_i_* changes were partially attributable to inaccurate AIF curve-fit extrapolations, the need for which arose from incorporating SOC imaging into our study design. A protocol using nonextrapolated image-derived AIFs or population-based AIFs might reduce these apparent temporal changes in *K_i_*. Several cases suggested late reversibility of DOTATATE binding, also contributing to the observed early-to-late *K_i_* decreases. Overall, cSUV_max_ and cSUR_max_ provided intrascan repeatability similar to that of *K_i_*_,max_, without dynamic imaging or AIF estimation.

*K_i_* images may still be worth their inherent complexities, as *K_i_* metrics appear useful for guiding treatment decisions and predicting oncologic outcomes (7*,*^8^*,*^25^*,*^26^). One study showed that *K_i_* correlated with SUR (*R*^2^, 0.96) much more strongly than with SUV (*R*^2^, 0.37), with all metrics measured at 50–60 min after injection (11). In contrast, we found that SUV_max_, cSUV_max_, SUR_max_, and cSUR_max_ all strongly correlated with *K_i_*_,max_ for both [^18^F]FDG (*R*^2^, 0.81–0.92) and DOTATATE (*R*^2^, 0.88–0.96), though cSUR_max_ had the best agreement with *K_i_*_,max_ across early and late time points for [^18^F]FDG (ICC, 0.69–0.75) and DOTATATE (ICC, 0.90–0.91). Our findings indicate that *K_i_* can be predicted from cSUR and that cSUR_max_ exhibits a nearly 1:1 proportionality to *K_i_*_,max_. To this point, cSUR and *K_i_* appear to predict postchemoradiation lung cancer outcomes better than does SUV (27). That said, Patlak images may provide higher lesion conspicuity and fewer false positives than with SUV images (28*,*^29^).

Our study has limitations, including its single-center, single-scanner design. The results should be corroborated at other centers on other scanners. Our patient cohort was heterogeneous; the relatively small sample size precluded subgroup analysis by cancer type or imaging indication. The *b* parameter of 0.63 for DOTATATE was derived empirically (rather than from AIF curve fitting) and needs to be validated in other cohorts. Again, the AIF curve-fit extrapolations created late *K_i_* errors. A more thorough investigation of potential causes of the observed temporal variability in *K_i_* is still warranted. Finally, our study excluded subjects who anticipated difficulty with a 90-min imaging period, potentially enriching our cohort for patients capable of remaining relatively motionless; as such, *K_i_* images may be more degraded by motion in an unselected oncologic population.

## CONCLUSION

*K_i_*_,max_, cSUV_max_, and cSUR_max_ exhibit comparably high intrascan repeatability in a general oncologic population undergoing PET with [^18^F]FDG or DOTATATE, with significantly less uptake time dependence compared with SUV_max_ and SUR_max_. cSUR_max_ can predict *K_i_*_,max_ without dynamic acquisitions.

## DISCLOSURE

This work was supported by a research grant from Siemens Healthineers to Washington University, including salary support for Tyler Fraum. Richard Wahl has received consulting income from Siemens Healthineers. All participants were imaged on a Siemens PET/CT scanner. Saeed Ashrafinia and Anne Smith are Siemens employees. These authors participated in the initial study design, provided occasional technical support, and critically reviewed the manuscript. However, all data collection, analysis, and manuscript preparation were performed by Washington University authors. No other potential conflict of interest relevant to this article was reported.

## Notes

KEY POINTS **QUESTION:** In oncologic PET, which quantitative metric of tracer avidity is least dependent on uptake times? **PERTINENT FINDINGS:** In this prospective, cross-sectional study of general oncology subjects undergoing PET with [^18^F]FDG or DOTATATE, *K_i_*_,max_, cSUV_max_, and cSUR_max_ exhibited comparably high intrascan repeatability, whereas SUV_max_ and SUR_max_ substantially increased at later uptake times. **IMPLICATIONS FOR PATIENT CARE:** Tracer-avidity metrics with high intrascan repeatability should be used to avoid quantitative interpretive errors related to different uptake times across scans.
